# The Relationship between the Migrant Population’s Migration Network and the Risk of COVID-19 Transmission in China—Empirical Analysis and Prediction in Prefecture-Level Cities

**DOI:** 10.3390/ijerph17082630

**Published:** 2020-04-11

**Authors:** Chenjing Fan, Tianmin Cai, Zhenyu Gai, Yuerong Wu

**Affiliations:** 1 College of Landscape Architecture, Nanjing Forestry University, Nanjing 210037, China; 2 School of Architecture, Tsinghua University, Beijing 100084, China; 3 Department of Health Care & Medical Technology, Nanjing Benq Medical Center, Nanjing 210037, China

**Keywords:** migrant population, COVID-19 transmission, Spring Festival travel rush, China, migration network, emerging epidemic

## Abstract

The outbreak of COVID-19 in China has attracted wide attention from all over the world. The impact of COVID-19 has been significant, raising concerns regarding public health risks in China and worldwide. Migration may be the primary reason for the long-distance transmission of the disease. In this study, the following analyses were performed. (1) Using the data from the China migrant population survey in 2017 (Sample size = 432,907), a matrix of the residence–birthplace (R-B matrix) of migrant populations is constructed. The matrix was used to analyze the confirmed cases of COVID-19 at Prefecture-level Cities from February 1–15, 2020 after the outbreak in Wuhan, by calculating the probability of influx or outflow migration. We obtain a satisfactory regression analysis result (*R*^2^ = 0.826–0.887, *N* = 330). (2) We use this R-B matrix to simulate an outbreak scenario in 22 immigrant cities in China, and propose risk prevention measures after the outbreak. If similar scenarios occur in the cities of Wenzhou, Guangzhou, Dongguan, or Shenzhen, the disease transmission will be wider. (3) We also use a matrix to determine that cities in Henan province, Anhui province, and Municipalities (such as Beijing, Shanghai, Guangzhou, Shenzhen, Chongqing) in China will have a high risk level of disease carriers after a similar emerging epidemic outbreak scenario due to a high influx or outflow of migrant populations.

## 1. Introduction

Coronavirus, widely distributed in humans, other mammals, and birds, can cause respiratory, intestinal, hepatic, and nervous system diseases [1]. Due to the difficulty in detection, the long incubation period, and the existence of asymptomatic carriers that can transmit the disease [2], coronavirus disease 2019 (COVID-19) quickly spread in China in late December in a very short period of time after its outbreak in early December of 2019 [3]. As of February 15, 2020, there have been 68,425 cases of COVID-19 infections confirmed in mainland China, including 1663 deaths.

The migrant population outflow from the outbreak center in immigrant Wuhan city, which accommodates 5.103 million migrants, is a factor that is responsible for the transmission of the disease to other regions. In China, there is a Spring Festival travel rush (“*chunyun*”) [4] every year, and migrants customarily return to their birthplace for the celebration. There is a 40-day period with a very large surge in transportation across China, which began on January 10, 2020 [5]. In the national transmission of COVID-19, the return of asymptomatic patients to their birthplace resulted in the widespread transmission of the disease, according to existing reports from the websites of health commissions. For example, in Harbin city, which is 2000 km away from Wuhan, the confirmed cases of COVID-19 infections that occurred before February 15 included 15 people who returned to Harbin from Wuhan. After that, the cluster of disease occurrences involved 111 cases, accounting for 79.3% of confirmed cases. To prevent COVID-19 from rapidly spreading with population movements, the Wuhan Municipal Government completely closed the city at 10:00 a.m. on January 23, 2020. Nevertheless, approximately 5 million people still left Wuhan, and large-scale migration activity may be an important cause of the long-term expansion of this disease. 

Networks and the epidemiology of directly transmitted infectious diseases are fundamentally linked [6]. Networks can be used to represent the patterns of connectivity of populations, and therefore describe aspects of disease transmission that depart from the mean field model [7,8,9]. This directional network transmission through intensified travel and migration renders political borders and geographical distance irrelevant and creates further possibilities for disease transmission [10,11,12,13,14], especially in the early disease growth phase. Network models usually show less variation and higher accuracy [15]. Existing research on the disease transmission network has predicted the transmission of a disease through small-scale population surveys [16] and traffic volume [17,18,19,20,21,22]. Part of the current research on COVID-19 also includes traffic forecasts from Wuhan [5]. However, the Chinese migration in the spring may involve other forms of transportation that are both public and private. Therefore, disease transmission cannot be predicted by a single transportation method. Recent related studies on COVID-19 are focused on predicting the number of people who have the disease [4,23,24,25]; in contrast, studies of the transmission and spatial distribution of COVID-19 in China are very scarce.

Through network research, prediction is essential to prevent the transmission of disease in new regions in the future. We asked the following two questions: (1) What are the consequences of similar emerging epidemic occurring in other immigrant cities? (2) How can policies for similar emerging epidemic scenarios be formulated? Based on these questions, we used the China Migrant Population monitoring data 2017 (CMDS 2017) (Sample size = 432,907) to construct a network called Residence place–Birthplace probability matrix (R-B matrix) to predict migrant influx and outflow migration. An empirical study was then performed using COVID-19 transmission as an example, and compared the advantages of using the R-B matrix with population or population density to predict disease transmission. Then, we simulated the outbreak scenario in the immigrant cities with the highest migrant population in China, and put forward some risk prevention measures after an emerging epidemic outbreak in the future.

## 2. Methods

### 2.1. Data

#### 2.1.1. China Migrant Population Dynamic Survey 2017 (CMDS 2017)

In 2017, the National Health and Family Planning Commission of China (NHFPC) conducted a CMDS throughout China. The sample points were distributed in 330 prefecture-level cities in 31 provinces. Interviewees were the migrant population residents who had lived in the inflow residence places for more than a month, did not have household registration in that city, and were at the age greater than 15 by 2017. Those whose current residence was different from the place of their household registration in the same city were not included. According to the survey plan, 170,000 domiciles were to be interviewed, and 169,989 were actually interviewed, involving 667,122 cohabited members, of which 575,288 cohabited members were in the inflow places. The monitoring included obtaining basic information on the migrant family, housing, basic public health, places of household registration, and so on. This survey includes the latest and widest data on the migrant population in China, and the results of the survey include well-represented urban groups and key cities.

#### 2.1.2. COVID-19 Infections Confirmed the Data from Prefecture-Level Cities

The confirmed disease data for prefecture-level cities came from the National Health Commission of China and provincial health commissions. We counted the number of confirmed cases in 330 prefecture-level cities during February 1–15, 2020, and this number reported by provinces in mainland China did not include Hong Kong, Macao, or Taiwan. Moreover, the data for the number of cases in these cities and the city names in the survey data for the CMDS 2017 were matched at the prefecture level. 

#### 2.1.3. Cofactors for Disease Transmission

The number of people infected in different prefecture-level cities will also be affected by cofactors. In addition to the CMDS data, we also incorporated the population data and population density of prefecture-level cities that is commonly used in traditional infectious disease network research as cofactors to indicate the infective host density and total host population [26,27]. These data were extracted from “China’s urban construction Statistical Yearbook 2019”.

### 2.2. Residence–Birthplace Probability Matrix (R-B Matrix)

First, we used the migrant population’s residence place and their household registration data from the survey data to construct the migrant Residence–Birthplace number matrix to model the migration network of migrants [28]. The residence place data were derived from the place of residence of each migrant population at the time of the survey, and the birthplace data were derived from the places of respondents’ household registration data, which refers to the place where Chinese residents enter their information into the household register, which is usually the place where their parents were registered at their birth. 

When we constructed the matrix, we deleted the following population samples: first, the local population that obtained a household registration; second, the population that lives elsewhere. By this method, the migrant population sample can well reflect the birthplace of a certain migrant population, with a total sample size of 432,907. The Residence–Birthplace number matrix formed is as shown in Equation (1):

(1) $$N=\left|\begin{array}{ccccc} n_{1,1} & \cdots & n_{1,j} & \cdots & n_{1,m}\\ \vdots & \ddots & & & \vdots \\ n_{i,1} & & n_{i,j} & & n_{i,m}\\ \vdots & & & \ddots & \vdots \\ n_{m,1} & \cdots & n_{m,j} & \cdots & n_{m,m} \end{array}\right|.\;$$

where $n_{ij}$ represents the number of residents born in node city *i* and living in node city *j* in the survey data; *m* denotes the number of cities, and there are a total of 330 cities in this study. Because the sample of the migrant population that obtained residence registration was excluded, the $n_{ab}$ in the matrix diagonal is 0 (Equation (2)).

Then, we standardized the probabilities of each residence place, adding up to 1 for each column or row, to form a probability matrix called the R-B matrix that illustrates how the migrant population in each city migrated from some birthplaces:

(2) $$P=\left|\begin{array}{ccccc} p_{1,1} & \cdots & p_{1,j} & \cdots & p_{i,m}\\ \vdots & \ddots & & & \vdots \\ p_{i,1} & & p_{i,j} & & p_{i,m}\\ \vdots & & & \ddots & \vdots \\ p_{m,1} & \cdots & p_{m,j} & \cdots & p_{m,m} \end{array}\right|$$

$$\mathrm{Note}:\;p_{i,j=}\;\{\begin{array}{c} \;p_{\overset{\leftarrow }{i,j}}=n_{i,j}/\overset{m}{\underset{j=0}{\sum }}n_{m,j}\;\\ p_{\vec{i,j}}=n_{i,j}/\overset{m}{\underset{i=0}{\sum }}n_{i,m} \end{array}$$

where $p_{\overset{\leftarrow }{i,j}}$ represents the probabilities of the migrant population residence in node city *i* whose birthplace was node city *j*.$\;p_{\vec{i,j}}$ represents the probabilities of the migrant population migrating to a residence in node city *j*, among the migrant populations whose birthplace was node city *i.* Taking Wuhan as an example, we can see that the variables related to the migration of migrants in Wuhan are $p_{\overset{\leftarrow }{wuhan,j}}$ and $p_{\vec{wuhan,j}}$ (*j* = 1 to *m*).

### 2.3. Correlation between the Transmission of the Disease and the Birthplace Probability of the Migrant Population by the R-B matrix

We initially considered constructing a model to verify whether the R-B matrix and cofactors can predict transmission of the disease at Prefecture-level Cities (Equation (3)).

(3) $$I_{a,t,j}=f\left(PopD_{j},\;Pop_{j},\;p_{\overset{\leftarrow }{a,j}},\;p_{\vec{a,j}}\right)$$

where $I_{a,t,k}$ is the number of confirmed cases in city *j* on day *t* after an outbreak occurred in city *a*. $PopD_{j}$ is the population density of *j* city and is used to indicate the infective host density, $Pop_{j}$ is the population of *j* city and is used to represent the host population [26,27], and $p_{\overset{\leftarrow }{a,j}}$ and $p_{\vec{a,j}}$ indicate the probability of migration direction between cities *a* and *j*, respectively (Equation (3)).

Using the number of diseases in prefecture-level cities 15 days after the outbreak of COVID-19 in 2020 as an example, the city *a* in the equation is Wuhan, *j* denotes the 329 prefecture-level cities in China except Wuhan, and *t* denotes the 15 days starting from February 1, 2020. We performed a bivariate correlation analysis between the $PopD_{j}\;,Pop_{j},\;p_{\overset{\leftarrow }{a,j}},\;p_{\vec{a,j}}$ with $I_{a,t,j}$ in prefecture-level cities during February 1–15, 2020. The Pearson correlation coefficient (*r*) was used to test these correlations and to determine which correlation was stronger. 

### 2.4. Prediction of Risk Levels in Prefecture-Level Cities and Similar Immigrant Cities and Outbreak Scenarios

(4) $$I_{a,t,j}=\beta_{1,t}\times PopD_{j}+\beta_{2,t}\times Pop_{j\;}+\;\beta_{3,t}\times p_{\overset{\leftarrow }{a,j}}+\beta_{4,t}\times p_{\vec{a,j}}$$

Based on the correlation analysis in Section 2.3, we used the indicators in Equation (3) that significantly affect $I_{a,t,j}$ to build a regression model and perform risk prediction. We assumed that the disease would occur in the largest 22 immigrant cities in China (each city has a migrant population of more than 2 million people, except for Wuhan) and predicted the risk of transmission to other prefecture-level cities using the same outbreak scenario on February 15, 2020 as that of Wuhan, and the risk level according to $I_{a,t,j}$ can be used to plot the risk map. We also calculated the standard deviation for the risk level at different outbreak scenarios for different prefecture-level cities. A smaller standard deviation value indicates that the disease transmission will be more extensive, and further attention will be required. 

Finally, because the location of the next emerging epidemic outbreak is uncertain, we also conducted an analysis of the highest risk of the 330 prefecture-level cities based on the risk map, and calculated the total risk values of other cities in the top 22 largest immigrant cities after the outbreak. By using the risk ranking of disease transmission in prefecture-level cities, it is evident that greater priority should be given to the formulation of emergency response policies in these cities.

## 3. Results

### 3.1. Construction of the R-B Matrix

**Figure 1:**
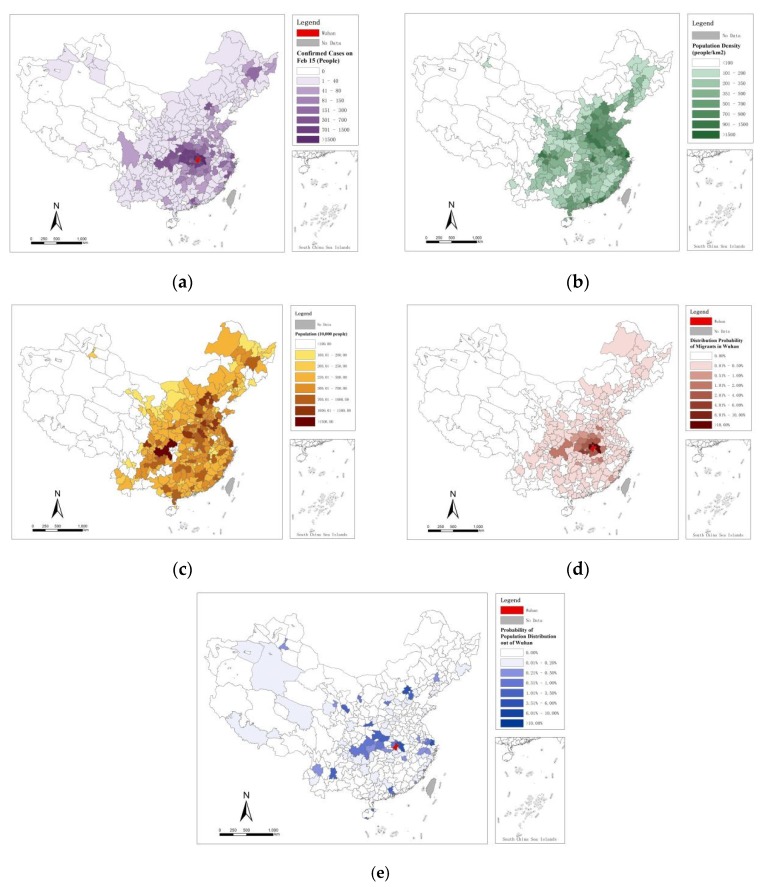
Prefecture-level city-related data. (**a**) $I_{Wuhan,t,j}$ in February 15, 2020; (**b**) $PopD_{j}$; (**c**)$\;Pop_{j}$; (**d**) $p_{\overset{\leftarrow }{wuhan,j}}$; (**e**)$\;p_{\vec{wuhan,j}}.$.

We used places of residence and household registration data from the CMDS 2017 data to construct an R-D matrix. The distribution of confirmed cases of COVID-19 infections ($I_{a,t,j})$, $PopD_{j}$, $Pop_{j}$, $p_{\overset{\leftarrow }{wuhan,j}}$ and $p_{\vec{wuhan,j}}$ at the prefecture level in China are shown in Figure 1.

### 3.2. Bivariate Correlation Test and Regression Results 

The Pearson correlation coefficients (*r*) for $PopD_{j}$, $Pop_{j},p_{\overset{\leftarrow }{wuhan,j}}$, $p_{\vec{wuhan,j}}$ were used to obtain Table 1 by bivariate correlation analysis with the number of COVID-19 infections confirmed $I_{Wuhan,t,j}$ in 15 days after the outbreak of the disease, in prefecture-level cities in China, and these 4 factors are significantly related to $I_{Wuhan,t,j}$. Among them, the correlation coefficient *r* of $p_{\overset{\leftarrow }{wuhan,j}}$ is the highest and rises to 0.918, which can prove that the birthplace probability of the migrant population in Wuhan is highly correlated with the number of confirmed populations in prefecture-level cities in China. In short, the $p_{\overset{\leftarrow }{wuhan,j}}$, and $p_{\vec{wuhan,j}}$ we focused from the R-B matrix can be used as a risk predictor in the prediction scenario.

After that, we constructed 15 regression models containing $PopD_{j}$, $Pop_{j}$, $p_{\overset{\leftarrow }{wuhan,j}}$, and $p_{\vec{wuhan,j}}$ to fit $I_{Wuhan,t,j}$ (Table 2). After a collinearity examination, we found that multicollinearity among all regression models was low, with variance inflation factors (VIF) of less than 1.5 [29,30]. It can be seen that in different models, $p_{\vec{wuhan,j}}$ and $p_{\overset{\leftarrow }{wuhan,j}}$ contributed more explanatory power, with the power of $p_{\vec{wuhan,j}}$ increasing day by day, which is completely consistent with our conjecture. People who work in Wuhan and return to their hometown are the main reason for the spread of the disease.

### 3.3. Results of Prediction of Risk Levels in Prefecture-Level Cities with Outbreak Scenarios in Similar Immigrant Cities 

**Figure 2:**
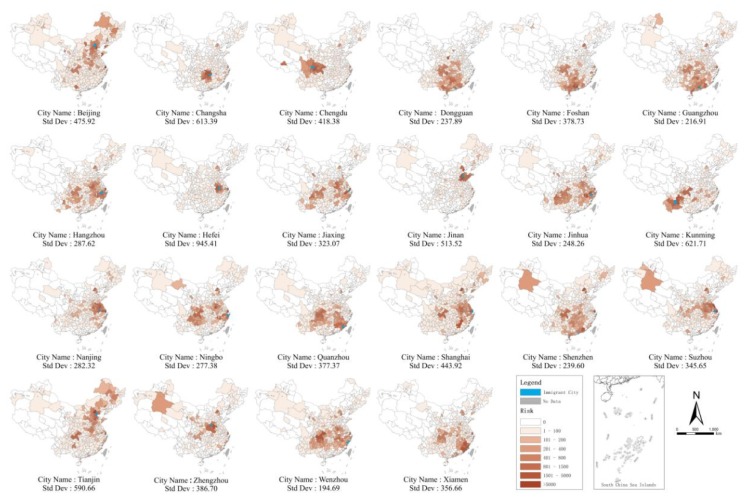
Risk ranking of other prefecture-level cities in the top 22 immigrant cities under a similar emerging epidemic outbreak scenario.

It can be seen that the model in Table 2 has good interpretation ability (*R^2^* = 0.826–0.887), and therefore, we will further use these models to the risk level according to $I_{a,t,j}$. Assuming that the time of the outbreak remained unchanged, Figure 2 shows the risk ranking of other prefecture-level cities after the outbreak of COVID-19 in the top 22 immigrant cities with the most migrant populations in China (due to space limitations, we only show the scene on February 15, 2020). According to the standard deviation index, when similar emerging epidemic outbreaks occur in Wenzhou, Guangzhou, Dongguan, and Shenzhen, the outbreaks will be more extensive in the affected cities, while those occurring in Kunming, Hefei, and Changsha will be more concentrated. 

### 3.4. Prefecture-Level City Risk Ranking of Disease Transmission 

**Figure 3:**
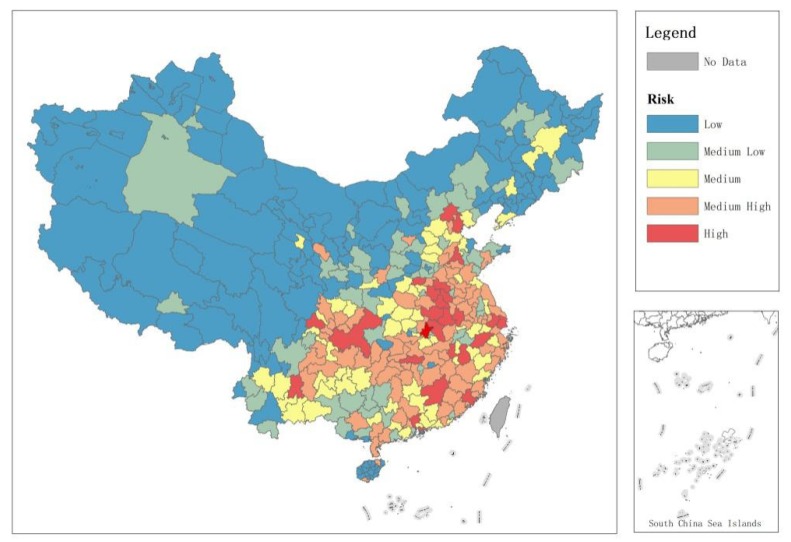
Total risk ranking of prefecture-level cities under a similar emerging epidemic scenario occurrence (reclassify by natural break).

According to the results of prediction of risk level in Section 3.3, the total risk values can be calculated for an emerging epidemic outbreak scenarios for Chinese prefecture-level cities in the top 22 largest immigrant cities. These prefecture-level cities at the highest risk after disease outbreak in immigrant cities are listed as Figure 3, which shows that cities in Henan province, Anhui province, and Municipalities (such as Beijing, Shanghai, Guangzhou, Shenzhen, Chongqing) have the highest risks. In contrast, Xizang, Qinghai, and Xinjiang have the lowest risk and smallest numbers of migrants, with populations that rarely migrate.

## 4. Discussion

### 4.1. COVID-19 Transmission in China is Highly Correlated with Factors that Return Migrants to Their Birthplace 

Most of the differential spatial transmission of COVID-19 in China is due to migrants returning from affected regions. Long-distance transmission of COVID-19 at this time, from cities such as Harbin and Wenzhou, is due to the large numbers of migrants from Wuhan in these cities and the probability that the migrants will engage in long-distance travel. A large number of studies used the number of transport networks to examine the possibility of transmission outbreaks. However, this method is inaccurate in predicting migrants’ homecoming in China. The transportation used for returning home in China in the spring may consist of multiple forms, including self-driving, which may be different from other forms of migration. Therefore, it is not feasible to use only a single transportation method to make predictions. 

China is a country undergoing rapid urbanization, and during this process, there is a large migrant population. In this study, we take advantage of the important characteristic of migration in the Spring Festival travel rush with a clear direction, that is, returning to the birthplace, and propose the idea of using the information of residence place and birthplace in the migrant population questionnaire to build a Residence place–Birthplace probabilities network. The bivariate correlation analysis and regression results in Table 1 and Table 2 show that this method can satisfactorily explain the spatial disease transmission mode, and why the disease does not occur in cities with high population and density in China. It can also simulate the risk and the number of confirmed diagnoses in other prefecture-level cities after the transmission of COVID-19.

### 4.2. Different Emergency Management and Control Plans for Different Disease Outbreak Scenarios in Immigrant Cities

Because of the characteristics of human networking, the transmission of disease is spatially heterogeneous. Spatially localized mass treatment is a crude approach compared to detailed contact tracing [31,32,33], but it may be implemented more quickly in practice. However, its broad nature poses problems: the number of individuals and regions affected by the intervention may be larger, with corresponding burdens that must be accommodated; if the intervention is harmful at the individual level (such as isolation), individuals and economic development will suffer unnecessary losses. It should be considered how to better integrate all focused interventions. Although a large number of recent reports have predicted how many people would contract pneumonia [4,23,24,25], there are few studies on the different threat levels of similar emerging epidemic for cities across the country, and the extent of this threat has not been quantified. In terms of COVID-19 (Figure 1), it is not appropriate for China to carry out nationwide intervention by stopping work and implementing school closures.

For cities not in the disease region, when there is a disease situation in other cities, we need to consider the origin of immigrants from different places by $p_{\overset{\leftarrow }{a,j}}$, and the probability of migrants in this city going to different cities by $p_{\vec{a,j}}$ through the R-B matrix. The use of the two sets of migration probabilities is helpful to formulate the post-disease spatially differential control policy. According to our forecast, as shown in Figure 2, in Kunming, Hefei, and Changsha cities, most of the migration originates from the province or nearby cities, which has little impact on other cities in China and is relatively simple to control. However, in Wenzhou, Guangzhou [34], Dongguan, Shenzhen, or other cities, most of the migrant population originates from different provinces that are distributed all over China. Therefore, there is a wide range of diffusion, and it has a large impact on other cities and is relatively difficult to control. In short, we believe that a minimum amount of risk management and control should be implemented for large-scale intervention, and we must implement different levels of emergency plans according to the risk of disease transmission.

### 4.3. Policy Intervention and Disease Prevention in the Important Migrant Birthplace Regions

Accurate geographic migration networks enable early and efficient planning of interventions to make the best use of scarce resources or manpower. We identified these high-risk node cities in Figure 3. On the one hand, most migrant birthplace regions tend to be economically and socially underdeveloped, with large birth populations, and are important nodes in the network of emerging epidemic [16,33]. By constructing a matrix, we identified these important migrant birthplaces by $p_{\overset{\leftarrow }{a,j}}$, and predicted the overall risk of transmission to other cities under the same circumstances if similar emerging epidemic scenarios occur in other large immigrant cities. The results in Figure 3 show that the regions with the highest probability are in the center of China, and these cities are located in Henan and Anhui provinces, which are economically underdeveloped but have large populations. On the other hand, some important migrant concentration node cities calculated through the index $p_{\vec{a,j}}$ are also worth noting. These cities are often world-class global cities with more developed economies and very close links with other immigrant cities in China. Figure 3 shows that these cities with high risk include Beijing, Shanghai, Guangzhou, Shenzhen and Chongqing. Therefore, in general, it is most effective to make emergency plans for these high-risk node regions under disease spread situations.

The following emergency plans need to be prioritized in these important node regions: (1) Plans for the deployment of vaccines, the provision of detection reagents, the provision of protective equipment (masks, disinfectants), and the provision of disease prevention personnel [17,33] will interfere with disease transmission through biosecurity measures. (2) Carrying out the construction of emergency isolation facilities (such as temporary infectious disease hospitals or reconstruction plans), plans for the control and isolation of populations returning to their hometowns, plans for suspension of school and work [1], and strict adherence to universal precautions in health care settings are critical in controlling the transmission of disease through isolation measures in these node cities.5. Conclusions

The outbreak of COVID-19 in China has attracted wide attention from all over the world. The impact of COVID-19 has been significant, raising concerns regarding public health risks in China and worldwide. A large migrant population left Wuhan and returned to their hometowns for the Spring Festival, which resulted in the transport of a large amount of virus to other regions. 

In this study, we constructed a matrix of the residence–birthplace (R-B matrix) of migrant populations by using the data from the China migrant population survey in 2017. This matrix was used to analyze the confirmed cases of COVID-19 from February 1–15, 2020 after the outbreak in Wuhan. Then, we used this R-B matrix to simulate an outbreak scenario in 22 immigrant cities in China, and proposed risk prevention measures after the outbreak. It was found that similar emerging epidemic scenarios would occur in the cities of Wenzhou, Guangzhou, Dongguan, or Shenzhen, with a wider disease transmission. We also used the matrix to determine that cities in Henan province, Anhui province, and Municipalities (such as Beijing, Shanghai, Guangzhou, Shenzhen, Chongqing) have a high risk level of disease carriers after the outbreak due to a high influx or outflow of people. Thus, we should give priority to formulating policies that will ensure that an emergency response plan is launched first after the occurrence of a similar emerging epidemic outbreak to check, control, and isolate people who leave or return after the outbreak. 

Our method has several limitations. First, the method in this study was only obtained from the perspective of the migrant population, but a small proportion of the registered population also returned to their birthplace during the Spring Festival after the outbreak of the disease. Second, COVID-19 was transmitted by cross-provincial, cross-city, and trans-national travel. In addition, inadequate criteria for early outbreaks may have contributed to some of the errors in the number of people diagnosed, and therefore, further research is needed. However, the migrant population contributed greatly to disease spread, and we believe that limiting migration and isolating people is one of the most effective ways to control an emerging epidemic. 

Our suggestions are very helpful and relevant, and would assist the Chinese government in its future management of disease. Priority should be given to research on the migration places of migrant populations in immigrant cities so that targeted policies can be formulated, especially population control during the Spring Festival. Simulation of the transmission of secondary infections in migrant birthplaces can also contribute to long-term infection research. In addition, it is necessary to model the number of people infected at different times.

In summary, this study provides a new perspective for the examination of epidemiological and behavioral data by proposing migrant networks. We have shown that we did not analyze traffic data (e.g., number of flights or train frequencies), but rather, we analyzed the social network causes of traffic migration. This is more predictive, and even in complex situations, simple tools can be designed to provide a method for predicting the transmission of disease and measuring risk. It is hoped that by providing simple and accurate approximations, a constantly evolving understanding of the complexity of the real world will be gained, and the method can continue to assist in understanding and controlling diseases. 

## Figures and Tables

**Table 1:** Results of bivariate analysis (*r*) of $PopD_{j}$, $Pop_{j}\;$,$p_{\overset{\leftarrow }{wuhan,j}}\;$, $\;p_{\vec{wuhan,j}}$ with $I_{Wuhan,t,j}$ during Feb 1, 2020 to February 15, 2020.

	$\mathrm{Pearson}\;\mathrm{Correlation}\;\mathrm{Coefficient}\;(r)\;\mathrm{for}\;I_{Wuhan,t,j}\;(N=329,t_{1}=\mathrm{February}\;1\;\ldots ,t_{15}=\mathrm{February}\;15)$														
	*t*_1_	*t*_2_	*t*_3_	*t*_4_	*t*_5_	*t*_6_	*t*_7_	*t*_8_	*t*_9_	*t*_10_	*t*_11_	*t*_12_	*t*_13_	*t*_14_	*t*_15_
$PopD_{j}$	0.157 **	0.146 **	0.142 *	0.136 *	0.129 *	0.128 *	0.128 *	0.127 *	0.126 *	0.125 *	0.125 *	0.120 *	0.117 *	0.117 *	0.117 *
$Pop_{j}$	0.232 **	0.220 **	0.212 **	0.200 **	0.187 **	0.182 **	0.177 **	0.175 **	0.173 **	0.172 **	0.170 **	0.162 **	0.158 **	0.155 **	0.154 **
$p_{\overset{\leftarrow }{wuhan,j}}$	0.836 **	0.849 **	0.857 **	0.883 **	0.907 **	0.915 **	0.917 **	0.915 **	0.915 **	0.915 **	0.916 **	0.913 **	0.915 **	0.916 **	0.918 **
$p_{\vec{wuhan,j}}$	0.543 **	0.534 **	0.514 **	0.500 **	0.477 **	0.466 **	0.463 **	0.461 **	0.458 **	0.456 **	0.453 **	0.447 **	0.445 **	0.439 **	0.436 **

**. Correlation is significant at the 0.01 level (2-tailed). *. Correlation is significant at the 0.05 level (2-tailed).

**Table 2:** Regression analysis of the $PopD_{j}$, $Pop_{j}$,$p_{\overset{\leftarrow }{wuhan,j}}$ and $p_{\vec{wuhan,j}}$ with the number of confirmed COVID-19 cases in prefecture-level cities $\left(I_{Wuhan,t,j}\right)$ from February 1, 2020 to February 15, 2020.

	$\mathrm{Model}\;\mathrm{for}\;I_{Wuhan,t,j}\;(N=329,t_{1}=\mathrm{February}\;1\;\ldots ,t_{15}=\mathrm{February}\;15)$														
	*t*_1_	*t*_2_	*t*_3_	*t*_4_	*t*_5_	*t*_6_	*t*_7_	*t*_8_	*t*_9_	*t*_10_	*t*_11_	*t*_12_	*t*_13_	*t*_14_	*t*_15_
	*Beta*	*Beta*	*Beta*	*Beta*	*Beta*	*Beta*	*Beta*	*Beta*	*Beta*	*Beta*	*Beta*	*Beta*	*Beta*	*Beta*	*Beta*
$PopD_{j}$	0.003	−0.003	0.003	0.008	0.017	0.024	0.029	0.030	0.031	0.031	0.034	0.034	0.034	0.038	0.040
$Pop_{j}$	0.119 **	0.108 **	0.102 *	0.082 *	0.063	0.056	0.049	0.048	0.047	0.046	0.044	0.036	0.032	0.029	0.027
$p_{\overset{\leftarrow }{wuhan,j}}$	0.715 ***	0.734 ***	0.751 ***	0.786 ***	0.823 ***	0.834 ***	0.837 ***	0.836 ***	0.836 ***	0.837 ***	0.838 ***	0.839 ***	0.842 ***	0.846 ***	0.849 ***
$p_{\vec{wuhan,j}}$	0.303 ***	0.295 ***	0.270 ***	0.250 ***	0.221 ***	0.206 ***	0.204 ***	0.202 ***	0.200 ***	0.198 ***	0.195 ***	0.191 ***	0.190 ***	0.183 ***	0.179 ***
*R* ^2^	0.826	0.835	0.836	0.865	0.881	0.887	0.887	0.883	0.882	0.880	0.880	0.883	0.885	0.885	0.887
*N*		329													

* *p* < 0.05; ** *p* < 0.01; *** *p* < 0.001.
